# Automated Brain Tumor Segmentation using Hybrid YOLO and SAM

**DOI:** 10.2174/0115734056392711250718201911

**Published:** 2025-07-30

**Authors:** Paul Jeyaraj M, Senthil Kumar M

**Affiliations:** 1 Department of Electrical and Electronics Engineering, Syed Ammal Engineering College, Ramanathapuram, 623502, Tamilnadu, India

**Keywords:** Image segmentation, CNN, YOLO v11, SAM, MRI, Brain tumor dataset

## Abstract

**Introduction:**

Early-stage Brain tumor detection is critical for timely diagnosis and effective treatment. We propose a hybrid deep learning method, Convolutional Neural Network (CNN) integrated with YOLO (You Only Look once) and SAM (Segment Anything Model) for diagnosing tumors.

**Methods:**

A novel hybrid deep learning framework combining a CNN with YOLOv11 for real-time object detection and the SAM for precise segmentation. Enhancing the CNN backbone with deeper convolutional layers to enable robust feature extraction, while YOLOv11 localizes tumor regions, SAM is used to refine the tumor boundaries through detailed mask generation.

**Results:**

A dataset of 896 MRI brain images is used for training, testing, and validating the model, including images of both tumors and healthy brains. Additionally, CNN-based YOLO+SAM methods were utilized successfully to segment and diagnose brain tumors.

**Discussion:**

Our suggested model achieves good performance of Precision as 94.2%, Recall as 95.6% and mAP50(B) score as 96.5% demonstrating and highlighting the effectiveness of the proposed approach for early-stage brain tumor diagnosis

**Conclusion:**

The validation is demonstrated through a comprehensive ablation study. The robustness of the system makes it more suitable for clinical deployment.

## 1 INTRODUCTION

Biopsies are used to classify brain tumors and can only be done through definitive surgery on the brain [1]. Physicians can utilize computational intelligence techniques to detect and categorize brain tumors. In this study, we suggested two deep learning techniques and incorporated YOLO with SAM strategies for identifying tumors, as well as healthy brains without tumors considered as normal, by analyzing magnetic resonance brain images to help doctors accurately detect tumors. In each of the grids of size S×S, an image is divided and extracted into m bounding boxes. In recent works, YOLO aims to solve a range of downstream segmentation problems on the brain tumor segmentation dataset. There are three are three main blocks in YOLO: backbone, neck, and head. The Resnet50 network acts as a backbone network for segmentation [2]. FPN is considered as neck part, which is used for multiscale fusion structure in the neck part. The feature selection using bounding box prediction is the head block. By training neural networks with CNN, various cell image characteristics can be understood, leading to powerful generalization capabilities [3]. The conventional CNN-based frameworks used are for segmentation tasks and their one-stage versions. Yet, these conventional approaches utilizing CNNs only reached less than ideal results for instant cell segmentation in real-time, particularly when working with crowded and diminutive cells [4].

The advanced level of segmentation and classification depends on the crucial and basic process of segmentation [5]. It helps medical professionals find brain cancer more easily. Processing of medical images involves analyzing images from an MRI or CT scanner. These datasets are utilized for guiding medical interventions like surgical planning. This analysis is used to acquire a comprehensive knowledge of a patient or groups within a Measurement, numerical assessment.

The MRI brain tumor (MRI-BT) images are first collected and pre-processed using the ResNet50 model, which is leveraged for its robust feature extraction capabilities. For efficient and rapid object detection, a combination of Feature Pyramid Network (FPN) and YOLO is employed. Following the bounding box predictions by YOLO, the Segment Anything Model (SAM) is applied to refine the segmentation, ensuring accurate lesion delineation without overlap based on input prompts like bounding boxes. The performance of the entire pipeline is evaluated using key assessment metrics such as precision, recall, and mean Average Precision at IoU threshold 0.50 (mAP50).

## 2 RELATED WORK

The YOLO series has become popular for its speed and accuracy in instance segmentation. Because of its one-stage design and feature extraction capabilities, YOLO models surpass two-stage segmentation models,

Yet, the capability of YOLO-based models to accurately segment small objects in image segmentation has not been fully explored. Segmenting cell instances is more difficult because of the presence of tiny, closely packed objects that overlap with one another, along with the unclear boundaries of cells, leading to potentially inaccurate segmentation. Accurately segmenting various objects in cell images in fine detail is necessary. because of variations in cell morphology, preparation techniques, and imaging tools [6]. Even though YOLO-based models show better accuracy and speed in segmenting natural images, there is still room for optimization when it comes to detecting small objects like cells in medical images.

Brain tumours can cause a range of symptoms [7]. Failure to correctly diagnose and address the lesion may lead to potential harm, severe disability, or fatality [8]. MRI is also capable of assessing. a patient's reaction to therapy and monitor BT progression throughout time. Furthermore, MRI has the capability to provide guidance. surgical procedure for removing a BT, with an emphasis on providing thorough information [9].

Unlike a U-Net, a Segmentation network (SegNet) conducts upsampling in its decoders by leveraging the pooling indices obtained during the max-pooling stages of the encoders [10]. Removing the requirement to learn parameters enhances boundary delineation and preserves more boundary information in the image representation, while also boosting inference efficiency in memory and computational time [11]. Yet, resulted in inaccurate object boundaries in its segmentation results in US images, attributed to degradations, limited large-scale datasets, and spatial information loss through downsampling. As a result, the SegNet's prediction can only be viewed as rough segmentation [12].

Segmenting them automatically is both difficult and essential. The complexity of MRI data from brain tumors comes from clinical scans or artificial databases [13]. The MRI scanner and methods employed can vary significantly between scans, leading to differences in intensity and other biases for each specific region in the dataset. This complexity is further heightened by the need for multiple modalities to thoroughly segment tumor sub-regions [14].

Brain imaging results are skewed because tumours are small in relation to the rest of the brain. Because of this characterisation, training a deep model frequently results in low true positive rates, and existing networks get biased towards the one over-represented class. Furthermore, the current deep learning methods require more time due to their intricate structures [15]. To get over the challenges, we have employed a potent pre-processing technique in our work that effectively eliminates a significant amount of unnecessary data, producing encouraging outcomes even with the current deep learning models [16]. Because of this approach, we can identify the tumour’s position and extract features without the need for a sophisticated deep learning model, which would have required a lengthy process [17]. Brain tumors are varied in their size, shape, location, and intensity, making the radiologist difficult even for distinguishing healthy tissues and tumor regions, especially during the early stage. The human errors, time consumption, image quality, presence of noise, and overlapping intensities complicate the diagnostic process. Recent advances in artificial intelligence (AI) have shown great promise in automating medical image analysis, particularly in effective brain tumor diagnosis. The system demands both precise localization and accurate delineation of tumor boundaries to help in treatment planning [18].

Additionally, the preprocessing phase in this technique reduces overfitting issues because the region of interest is smaller.. The use of MRI scans for automatic BT detection can greatly enhance diagnosis, treatment, and growth. Segmentation's goal is to identify the tumour area and outline the borders of its compartments: necrotic tissue, actively growing tissue, and edema (growth around the tumor). Mask R-CNN [18] enhances Faster R-CNN [19-22] by including region of interest (ROI) alignment procedures in two-phase methods. The combination of YOLO and CNN in Box Inst [23-28] enhances network efficiency.

The integration of YOLO with segment anything model (SAM) architectures like U-Net helps in achieving high precision in segmenting tumors within complex structures of the brain. The bounding boxes from YOLO allow CNNs to focus on ROIs, reducing the computational burden and enhancing segmentation quality within detected areas. YOLO has been used for brain segmentation to detect regions of interest (ROIs) that may contain tumors or other abnormalities, providing bounding boxes around these areas as a preliminary detection step.

## 3 MATERIALS AND METHODS

### 3.1 Image Preprocessing

Image preprocessing is crucial in diagnostic tasks since it improves image quality and readies them for accurate and effective diagnosis. The MRI images are preprocessed to enhance image quality, standardize input dimensions, and improve model performance. The preprocessing pipeline includes the normalization, where the MRI images are normalized to a [0,1] range to reduce the contrast effects, then gaussian filtering is applied to minimize the impact of random noise, and then resizing to a fixed resolution of 512×512 pixels to ensure the consistency for the input requirements. In this work, contrast enhancement is done through histogram equalization to improve the contrast between tumor regions and healthy brain tissues. Data augmentation is applied to prevent overfitting and increase model robustness during the training phase. The brain tumor yolov11 dataset contains MRI scans with different gaps in all three dimensions. The input images are down-sampled from 512×512×3 to 224×224×3. The image is enhanced by using FPN, which creates segmentation masks by combining high-resolution feature maps with strong semantic information. This results in better-defined boundaries for detected objects and enabling multi-scale feature representation without significant additional computation. During the initial processing step of the suggested method, every volume is trimmed to eliminate the unnecessary background, resulting in a reduction in computational workload. Next, for every slice, except for the ground truth, the pixel intensity at the top and bottom is limited to one percent. During the segmentation stage, any regular slices are not taken into consideration. The modalities are represented on axis 0 and the slice on axis 1 by swapping the axes. All pieces are shuffled in a random manner. YOLO takes a fixed-size input image (640X640). Figs. (**1** and **2**) shows the architecture of SAM and the system model. It comprises two input prompts and an image to accept bounding boxes as prompts to guide segmentation. The lightweight mask decoder helps to generate high resolution output around the image to enhance the tumor boundaries. The system model shows a framework for brain tumor detection and segmentation. The image first passes through a CNN with a ResNet50 backbone, and the architecture is divided into 5 stages with convolutional blocks, residual blocks, and identity blocks, ending with pooling and fully connected layers. YOLOv11 shows a feature pyramid Network (FPN) with multi-scale feature representations, and the final prediction block is shown with brain tumor detection and normal healthy images.

**Fig. (1):**
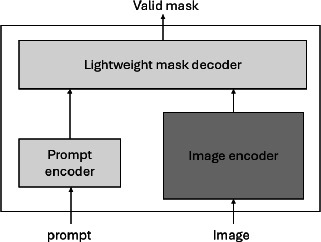
Segment Anything Model (SAM).

The architectural block of SAM is given in figure 1, which consists of a prompt and image encoders and a mask decoder to focus on boundaries and shape more accurately within the region of interest. These feature characteristics undergo a sequence of encoding and decoding layers to extract advanced semantic information and maintain spatial details.

The structure of the system model is shown in Fig. (**2**) , Firstly, the traditional convolutional network as ResNet50, is followed by the FPN to detect complex image features, YOLO block to predict clear boundaries to classify the brain tumor image.

### 3.2 Histogram Equalization

Histogram equalization is utilized to enhance the visibility of an image that contains several prominent intensity peaks in its histogram. An input probe image I is given with its pixel positions, intensity values, and intensity levels.

#### 3.2.1 Global Histogram Equalization

GHE is extremely easy and quick, although its ability to enhance contrast is limited. The histogram transformation function is calculated using the histogram of the entire input image. Consequently, the dynamic range is both flattened and stretched. Overall, there is an enhancement in contrast.

The probability of occurrence of grey level in an image is approximated by (Eq. **1)**

**Table:** 

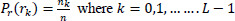	(1)

The discrete version of the transformation is given in (Eq. **2**)

**Table:** 

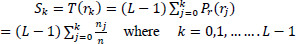	(2)

### 3.3 CNN Feature Extraction (Backbone Network)

An extraction of features is approached with ResNet50. There are 50 layers in the Resnet 50 to extract features. These feature maps are crucial for identifying objects and, in segmentation-based YOLO variants, for segmenting objects as well. ResNet50 trained on large datasets achieves state-of-the-art results. The output of ResNet50 was calculated using (Eq. **3**)

**Table:** 

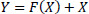	(3)

Y is the output and X is the input.

The first layer of Resnet is given in (Eq. **4**)

**Table:** 

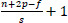	(4)

Where *n* × *n* is the image size, p is the padding size, and f is the filter size.

The initial convolutional layer receives a 224x224x3 image as input 112x112x64 feature map as output. The input image is divided into half in both width and height at this stage by applying 64 filters, each with a size of (7, 7) and a stepping of (2, 2). Following the convolutional layer, the activations are normalised by a batch normalisation layer. Nonlinearity is then introduced using a ReLU activation function. The feature map is then divided in half in both dimensions using a max pooling layer, which employs a stride of (2, 2) and a pool size of (3, 3). The feature map of size (56, 56, 64) is fed into the input of a convolutional block in the ResNet50 design.

The YOLO model's neck is the FPN; in this neck framework, features are mapped using a 3x3 convolution filter, and then bounding box prediction and regression are performed using a 1x1 convolution filter. The same layers are applied to map the multi-level feature segmentation and regression. The ReLu is an activation function to reduce the spatial dimensions.

### 3.4 Bounding Box Prediction (Detection Part)

The image is split into a S×S times, S×S grid in traditional YOLO. YOLO forecasts several bounding boxes, their confidence ratings, and class probabilities for every grid cell. Anchor boxes are used by the model to assess the size and form of objects that are detected. The output will contain details about the discovered objects, such as their class labels, confidence ratings, and bounding box coordinates, which can be obtained using YOLOv11. After that, you can identify and create bounding boxes around the identified items in the image by extracting the coordinates from the output.

This enhances the model's comprehension of the images, leading to a higher level of accuracy in object recognition. The final layers of the YOLO model consist of the Convolution (Conv) and Concatenation (Concat) layers. The Convolutional layers help adjust the features, and the Concatenation layers merge feature maps of different scales to form precise object descriptions.

### 3.5 Segmentation Mask Prediction (Segmentation Adaptation)

For segmentation, an additional branch is added to predict segmentation masks for each detected object. A pixel-wise classification layer is typically added to identify which pixels in the bounding box belong to the object (i.e., segmentation mask generation). CNN layers may be used here to extract fine-grained features for pixel-level segmentation.

#### 3.5.1 Prediction Refinement using SAM (Post-processing)

The primary difficulty is acquiring and preparing the data. Creating a dataset for image segmentation requires labeling many images, which is a huge undertaking. This requires a lot of resources. As a result, the game changed once the Segment Anything Model (SAM) was introduced. SAM enabled individuals to create segmentation masks for their data without relying on annotated data, revolutionizing this field.

#### 3.5.2 Mask Refinement

In the segmentation version, the predicted masks are further refined by adjusting the boundaries and ensuring alignment with the bounding box predictions. The image is encoded into vectors by the SAM component. These vectors depict the edges and outlines at a lower level, as well as the object shapes and textures at a higher level that have been extracted from the image. The Vision Transformer (ViT) used as the encoder here offers several benefits compared to conventional CNNs.

The mathematical equations in YOLO for estimating the bounding boxes involve calculating the width and height as distances from cluster centroids. The summary of the steps is as follows,

**Stage 1: Feature Extraction**

The MRI image is pre-processed, and the deep convolutional layers extract low-level and high-level features about the patterns.

**Stage 2: Real-Time Object Detection with YOLOv11**

The region of interest (ROI) is detected through YOLOv11 as the extracted feature maps are passed, and the anchor-based bounding box predictions enable accurate localization of unshaped tumors.

**Stage 3: Fine-Grained Segmentation with SAM**

The localized ROI is further processed by the SAM, where it performs pixel-wise segmentation within each detected ROI, generating detailed masks that delineate the exact boundaries.

**Stage 4: Integrated Prediction**

The detection results from YOLOv11 and the segmentation masks from SAM are integrated to produce a unified output.

## 4 EVALUATION AND ASSESSMENT CRITERIA

Precision, Recall, and Accuracy are the utilized metrics for assessing performance and will be elaborated on in the following sections.

### 4.1 Confusion Matrix

The metric offers an understanding of the accuracy and errors in the suggested models. The confusion matrix makes it possible to determine both the number of errors and their location (false positives versus false negatives). When the cost of several error types varies, this becomes crucial. In medical diagnosis, for instance, a false negative (missing a tumour) could be more dangerous than a false positive (diagnosing a tumour erroneously).

### 4.2 Loss

The loss function is formulated as in Eq. (**5**),

**Table:** 

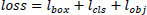	(5)

Where *l_box_* is the bounding box regression, which is described as (Eq. **6**)

**Table:** 

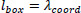	(6)

**Table:** 

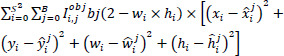	(7)

**Table:** 

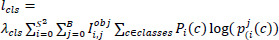	(8)

The mean square error is given in Eq. (**7)**, and the bounding box class loss function is calculated using (Eq. **8**).

The variables represent the target's real centre coordinate, whereas ˆ w and h represent the target's width and height, respectively. If there are targets in the anchor box at location (i,j), the associated value I_obj_ i,j is set to 1; if not, it is set to 0.Pl(C) is the actual category value, while pi(C) indicates the target's likelihood of falling into a particular category. Both variables' lengths add up to the total number of categories C.

The CNN integrated with Yolov11 segmentation outputs are shown in Fig. (**3a**, and **3b**). CNN layers analyze the MRI image, recognizing intricate features like edges, textures, and patterns that could signify areas of abnormality. These layers assist the model in understanding which features could indicate regions of interest in a medical image. YOLOv11 for Object Detection and Segmentation: it appears that two bounding boxes (marked in yellow) have been detected, probably relating to regions with unique features, like possible lesions or irregularities. Each box probably contains a related confidence score that reflects the likelihood of an abnormal area being present. The segmentation features include bounding boxes encasing the identified areas, enabling medical professionals to concentrate on zones. The highlighted areas, illuminated in white, probably signify regions that the model identifies as significant, potentially for additional examination or assessment. Figs. (**4** and **5**) illustrate the segmentation output after being combined with SAM for more predicted and accurate results. SAM is intended to concentrate on boundaries and define shapes more precisely within the area of interest. As illustrated in the segmented image to the right, SAM covers the tumor area with a yellow overlay, indicating the precise shape and extent of the tumor instead of merely a bounding box. This offers a clearer and more precise depiction of the tumor's dimensions and form.

Fig. (**6**) is shown after the MRI images were identified as a tumor with accuracy using the proposed system model. The collection is the BT segmented output from CNN+YOLOv11, which gives an approximate bounding box around the tumor in the original image, helpful for swiftly pinpointing the tumor's position. SAM, on the other hand, advances by offering detailed segmentation within this region, as demonstrated in the segmented image. Figs. (**7** and **8**) are the defined performance metrics of the model while using YOLOv11 and after the implementation of SAM.

### 4.3 Distributed Focal Loss (DFL)

DFL aims to enhance YOLOv11 by focusing on difficult detections, resulting in a significant improvement. Conventional loss functions may not capture intricate scenarios, whereas DFL guarantees that these challenging cases are given higher priority in the training process. The emphasis of the loss function in Eq. (**9**), is on objects that are challenging to detect, enhancing the precision given in Eq. (**10**).

**Table:** 

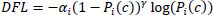	(9)

Where *α_i_* is the weighting factor to offset the class imbalance of the number of classes, and *γ* is the hard control parameter to adjust the focal length.

### 4.4 Precision

The performance metric to assess the correctness of the bounding box predictions is computed as follows

**Table:** 

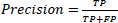	(10)

As TP and FP are true and false positive values.

### 4.5 Recall

The ratio of TP detections over the total TP and FN is computed as a recall metric and is given in Eq. (**11**),

**Table:** 

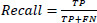	(11)

As FN is the false negative value.

### 4.6 Mean Average Performance (mAP50-95(B))

The mean average precision was calculated across different IoU thresholds, from 0.50 to 0.95 (Eq. **12**), to determine the average. It provides a thorough understanding of how well the model performs at varying levels of detection difficulty.

**Table:** 

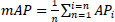	(12)

Where the number of classes is represented as n and *AP_i_* is the average precision of class i.

The exceptional precision and recall metrics achieved by the proposed hybrid CNN + YOLOv11 + SAM model have profound implications for clinical practice, particularly in the context of brain tumor diagnosis and treatment planning. High precision indicates that it is highly likely to be a true positive and not a false alarm, whereas high recall ensures that all tumors present in the MRI are almost detected by the system, minimizing false negatives.

## 5 RESULTS AND DISCUSSION

The part covers the conversation and execution of the suggested YOLOv11+SAM for the segmented result. The tests were conducted using the NVIDIA GeForce 3090 (24G) GPU along with PyTorch 3.9, Python 3.12.1and CUDA. The data is divided into three categories: training set, testing set, and validation set. 80% of the images are considered for training and validation, remaining 20% of the images are taken for testing. The dataset that is available to the public is sourced from universe.roboflow.com. CNNs' image is preprocessed by capturing global context information without adding to computational workload or parameter count.

YOLOv11 is optimized for real-time object detection and outputs bounding boxes with labels. SAM excels in segmentation tasks, producing detailed masks and working well with varied prompts to segment anything. The hyper parameters used in this segmentation are given in Table **1**.

The proposed model achieved a good recall rate with 95.6% through Precision as 94.2% and mAP of 96.5 while we fix the IoU threshold value as 0.5. The same model without SAM produces recall rate with 94.6% through Precision as 92.2% and mAP of 94.5 while we fix the IoU threshold value as 0.5. The comparative analysis report is presented in Tables **2** and **3**. Table **2** is the comparison between various YOLO models Table **3** is the comparative analysis report of various related works in terms of the performance parameters.

This results implementation of YOLO combined with SAM produces good results in terms of BT detection. The capacity of the model to locate and accurately classify objects within images is shown by a greater mAP and recall. This is known as detection performance (using YOLO). The bounding boxes of YOLO provide a precise foundation for SAM's segmentation. Segmentation Quality (using SAM): SAM's capacity to create pixel-accurate masks around detected objects is reflected in high IoU and Dice scores.

The proposed model is used for fine-tuning feature extraction by using CNN, YOLO for fast object detection, and SAM for detailed segmentation, which is especially useful in medical applications for visualizing exact tumor boundaries. The detection of BT during the testing is given in the confusion matrix and is shown in Fig. (**9**). The results are evident that the proposed model provides the precision and recall in the confidence curve, which are shown in Figs. (**10** and **11**)

The accuracy of YOLO's detection determines the segmentation quality because SAM obtains bounding boxes from YOLO. Accuracy and Speed Trade-offs: FPS measurements reveal information about the combined pipeline's delay. High FPS is advantageous for real-time applications; however, because segmentation is computationally expensive, adding SAM may result in lower FPS. The confusion matrix is given in Fig. (**9**) to show the performance of the classified image with tumors and normal. Recall confidence and precision confidence of the model are given in Figs. (**10** and **11**) to show the effectiveness of the model. The comparative performance analysis with other recent YOLO models is given in Table **2**, and with comparative performance results are given in Table **3**. The training loss and validation loss are detailed in Table **2**. The loss is specific for bounding box regression, segmentation masks, objectness score, and classification.

An evaluation of possible obstacles in applying the model to various datasets, along with different imaging platforms, is included in the Table **4** demonstrates the key features of the publicly available datasets. Table **5** demonstrates the ablation study with performance analysis, which helps to achieve significant benefits and validate architectural decisions with empirical evidence.

## 6 STUDY LIMITATIONS

YOLO is primarily designed for real-time object detection, and its performance depends heavily on the quality and nature of the images it processes.

Its success with MRI images (especially for tumor detection) comes from the ability of YOLO to recognize patterns and shapes. However, CT and PET scans present unique challenges like density differences and increased metabolic activity.

## CONCLUSION

The hybrid CNN+YOLO+SAM model is implemented in this study demonstrates a strong and effective framework to support a clinical tool for early brain tumor analysis, as evidenced by its accuracy, high precision, and recall metrics. This hybrid architecture addresses key challenges in tumor detection by combining the strengths of feature extraction, object detection, and advanced segmentation techniques, enhancing diagnostic capabilities to distinguish tumors and normal healthy tissues. Ablation study and comparative dataset analysis results show meaningful improvement over existing approaches. Future research should focus on improving model generalizability across diverse patient populations and MRI acquisition protocols.

## Figures and Tables

**Fig. (2):**
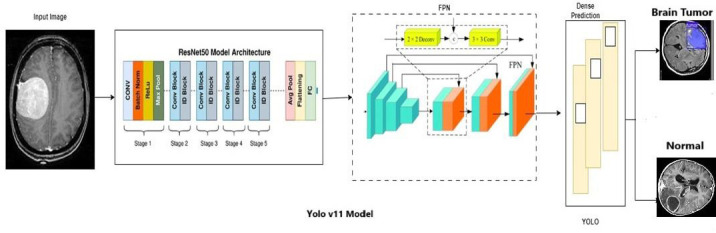
System model.

**Fig. (3a):**
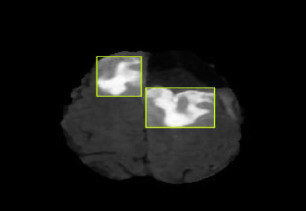
CNN+YOLO segmentation output.

**Fig. (3b):**
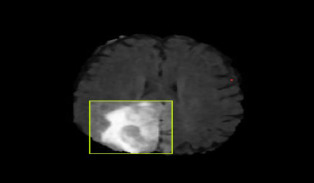
CNN+YOLO segmentation output.

**Fig. (4):**
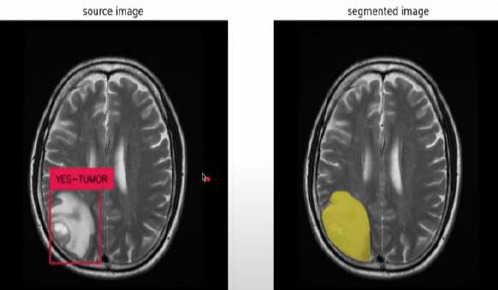
CNN+YOLO+SAM segmentation output.

**Fig. (5):**
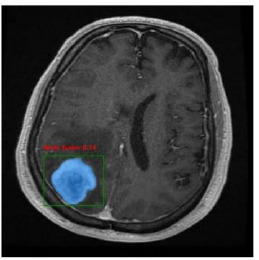
CNN+YOLO+SAM segmentation.

**Fig. (6):**
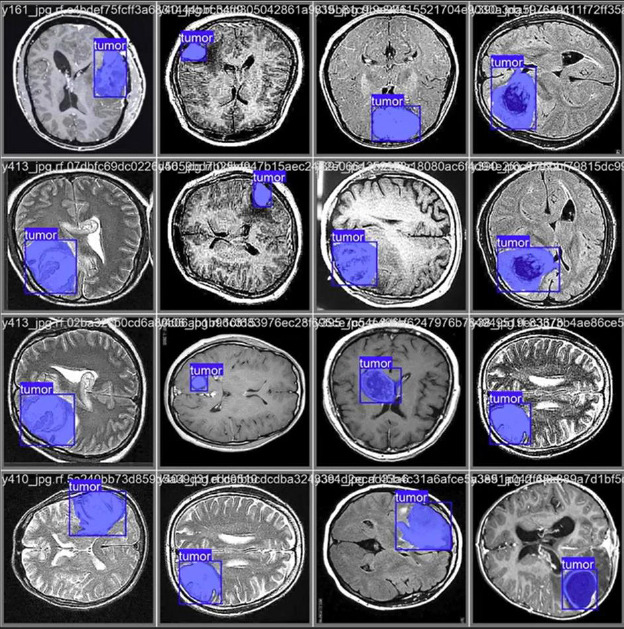
Brain tumor segmentation with accuracy.

**Fig. (7):**
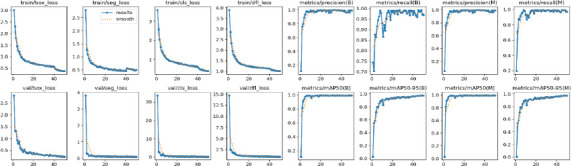
Performance metrics of CNN+YOLO.

**Fig. (8):**
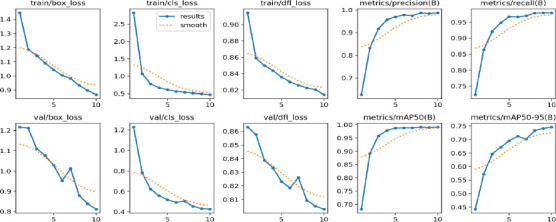
Performance metrics of CNN+YOLO+SAM.

**Fig. (9):**
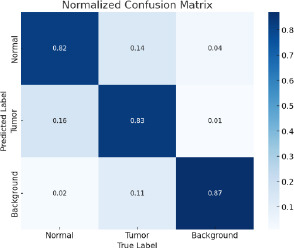
Confusion matrix of the proposed model.

**Fig. (10):**
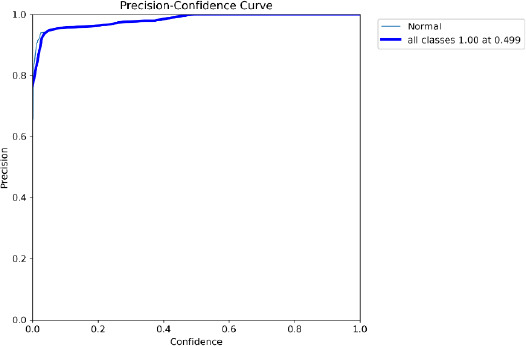
Precision confidence curve.

**Fig. (11):**
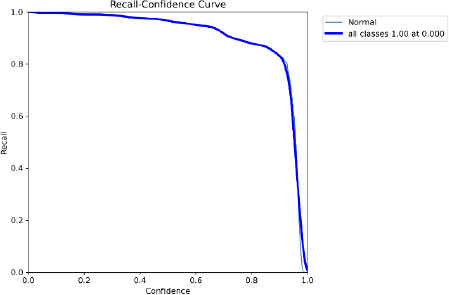
Recall confidence curve.

**Table 1:** Hyper parameters for the proposed model.

-	**Value**
**CNN+YOLO**	-
Learning rate	0.001
Momentum	0.92
Weight decay	0.01
Batch size	100
Epochs	200
Image size	640 × 640
IoU threshold	0.5
Confidence threshold	0.25
Dropout	0.1
**SAM**	-
Optimizer	AdamW
Learning rate	0.0001
Batch size	16
Epochs	100
Patch size	16 × 16
Mask threshold	0.5

**Table 2:** Comparative performance analysis with other YOLO models.

**YOLO**	**T/box**	**T/seg**	**T/obj**	**T/cls**	**V/box**	**V/seg**	**V/obj**	**V/cls**
v5	0.0176	0.0174	0.0056	0.0003	0.0257	0.0334	0.0050	0.0021
v7	0.0121	0.0142	0.0042	0.0003	0.0245	0.0365	0.0052	0.0018
v11	0.0113	0.0123	0.0040	0.0003	0.0242	0.0368	0.0054	0.0017

**Table 3:** Comparative performance analysis of the reference work.

**Contributors/Refs.**	**Proposed Model**	**Dataset used**	**Mechanism**	**Performance Metrics**
Asad R, Rehman, *et al*., 2023 [4]	DNN with stochastic gradient descent (SGD) optimization algorithm	Public Kaggle brain-tumor dataset	Feature extraction and classification	Training Accuracy- 99.82% Testing Accuracy 99.5%
Cai X, *et al*., 2021 [8]	A combined gray-level cooccurrence matrix (GLCM) and discrete wavelet transform (DWT) method	MIAS database	Segmentation	Accuracy – 93.79% sensitivity – 96.89% specificity – 67.7%
A.Hossain, *et al*., 2022 [29]	DL with YOLOv5	Microwave brain images	Classification	Accuracy – 95.2% mAP – 94.3% Precision – 95.2%
T.Liu, *et al*., 2021 [17]	CNN with sparrow search algorithm	BT dataset	Segmentation	Accuracy – 94.76% Recall – 97.2% Specificity – 67.8%
M.A.Almufareh, *et al*., 2024 [26]	CNN+YOLO v5 and v7	Figshare Dataset	Classification	Precision – 93.7% Recall – 90.6% mAP@.5 – 94.2%
Proposed Model	CNN+YOLO+SAM	Figshare Dataset	Classification	Precision – 94.2% Recall – 95.6% mAP50(B)– 96.5%

**Table 4:** Comparative model evaluation using different brain tumor datasets.

** Dataset **	** Source **	** Number of Images **	** Tumor Types **	** Key features **
BraTS 2021	BraTS Challenge	1000 MRI scans	Gliomas (high and low grade)	Multi-modal MRI scans, tumor annotations
ISLES 2017	ISLES Challenge	50-60 3D MRI scans	Ischemic stroke lesions	Focus on ischemic stroke lesions, detailed annotations, high-quality MRI
Brain MRI images for brain tumor detection	Kaggle	300 MRI images	Tumor *vs.* non-tumor (benign/malignant)	MRI images are labeled as tumor/non-tumor, binary classsification
** Proposed dataset ** ** roboflow BT **	universe.roboflow.com.	896 MRI images	Tumor *vs.* non-tumor (benign/malignant)	Detailed annotations, Roboflow provides ** collaborative features ** ,high-quality MRI

**Table 5:** Ablation study with performance results.

** Experiment **	** Model configuration **	** Object Detection Accuracy (%) **	** Segmentation Accuracy **	** Remarks **
1	Baseline CNN (ResNet 50)	88.7	88.4	Basic feature extraction, poor localization and segmentation
2	CNN+YOLOv11(without SAM)	92	89	Strong object detection but segmentation is not precise
3	CNN+SAM (Without YOLOv11)	91.8	92	Good segementation but weak ROI
4	CNN+YOLOv11+SAM	93.8	91.6	Best detection and segmentation performance

## Data Availability

The data supporting the findings of the article is available in the Roboflow Brain Tumor Detection and Figshare Dataset repository at: https://universe.roboflow.com [28, 29].

## LIST OF ABBREVIATIONS

AI: Artificial Intelligence
BT: Brain Tumor
CNN: Convolutional Neural Network
CT: Computed Tomography
DFL: Distributed Focal Loss
FN: False Negative
FP: False Positive
FPN: Feature Pyramid Netwok
GHE: Global Histogram Equalization
GPU: Graphics Processing Unit
IoU: Intersection over Union
mAP: Mean Average Precision
MRI: Magnetic Resonance Imaging
PET: Positron Emission Tomography
ResNet: Residual Network
R-CNN: Region based Convolutional Neural Network
ROI: Region of Interest
SAM: Segment Anything Model
TN: True Negative
TP: True Positive
SegNet: Segmentation Network
ViT: Vision Transformer
YOLOv11: You Only Look Once Version 11
